# The development of a theory-based intervention to promote appropriate disclosure of a diagnosis of dementia

**DOI:** 10.1186/1472-6963-7-207

**Published:** 2007-12-19

**Authors:** Robbie Foy, Jillian J Francis, Marie Johnston, Martin Eccles, Jan Lecouturier, Claire Bamford, Jeremy Grimshaw

**Affiliations:** 1 Institute of Health and Society, Newcastle University, 21 Claremont Place, Newcastle upon Tyne NE2 4AA, UK; 2 Health Services Research Unit, Health Sciences Building, University of Aberdeen, Foresterhill, Aberdeen AB25 2ZD, UK; 3 School of Psychology, College of Life Sciences and Medicine, William Guild Building, University of Aberdeen, Aberdeen AB24 2UB, UK; 4 Clinical Epidemiology Program, Ottawa Health Research Institute, 725 Parkdale Avenue, Ottawa ON K1Y 4E9, Canada

## Abstract

**Background:**

The development and description of interventions to change professional practice are often limited by the lack of an explicit theoretical and empirical basis. We set out to develop an intervention to promote appropriate disclosure of a diagnosis of dementia based on theoretical and empirical work.

**Methods:**

We identified three key disclosure behaviours: finding out what the patient already knows or suspects about their diagnosis; using the actual words 'dementia' or 'Alzheimer's disease' when talking to the patient; and exploring what the diagnosis means to the patient. We conducted a questionnaire survey of older peoples' mental health teams (MHTs) based upon theoretical constructs from the Theory of Planned Behaviour (TPB) and Social Cognitive Theory (SCT) and used the findings to identify factors that predicted mental health professionals' intentions to perform each behaviour. We selected behaviour change techniques likely to alter these factors.

**Results:**

The change techniques selected were: persuasive communication to target subjective norm; behavioural modelling and graded tasks to target self-efficacy; persuasive communication to target attitude towards the use of explicit terminology when talking to the patient; and behavioural modelling by MHTs to target perceived behavioural control for finding out what the patient already knows or suspects and exploring what the diagnosis means to the patient. We operationalised these behaviour change techniques using an interactive 'pen and paper' intervention designed to increase intentions to perform the three target behaviours.

**Conclusion:**

It is feasible to develop an intervention to change professional behaviour based upon theoretical models, empirical data and evidence based behaviour change techniques. The next step is to evaluate the effect of such an intervention on behavioural intention. We argue that this approach to development and reporting of interventions will contribute to the science of implementation by providing replicable interventions that illuminate the principles and processes underlying change.

## Background

Clinical and health services research is continually producing new findings that can contribute to effective and efficient health care. However, despite the considerable resources devoted to this area, a consistent finding is that the transfer of research findings into health care practice is unpredictable and can be a slow and haphazard process [1].

Ideally, the choice of implementation strategies would be based upon evidence from randomised trials [2]. Healthcare practitioners and managers should be able to read a systematic review of an implementation intervention, reliably replicate some – or all – of the components of successful interventions in their own settings, and be confident of what will happen as a consequence. However, this is not currently the case. This is largely due to a combination of the manner in which trials are reported and, at least partially as a consequence, the lack of detail included in reports of systematic reviews.

Systematic reviews of implementation trials conducted to date have categorised interventions on an empirical basis with reviews of interventions such as audit and feedback, reminders, and outreach visiting [3]. Such classification systems appear to be largely based on intuitive principles, somewhat akin to classifying drug interventions on the basis of whether the drugs are taken orally or intravenously. It is subsequently not surprising that systematic reviews based on these categories raise more questions than they answer. Indeed, reviews of implementation interventions produce a consistent message – all interventions, both within and across categories, are effective some but not all of the time, producing a range of effect sizes from no effect through to a large effect. The substantial heterogeneity of intervention components, targeted behaviours, and study settings make generalising findings from these studies to routine healthcare settings problematic. There is no underlying generalisable taxonomy by which to characterise these interventions, targeted behaviours and settings. There is only a limited and sometimes hopeful understanding of the 'active ingredients' required to develop a successful implementation strategy [4,5].

One way of addressing a situation such as this is to tackle the issue empirically by examining all relevant combinations of the perceived important and modifiable elements of interventions to determine which contribute to a successful intervention. For example, audit and feedback has a range of modifiable elements which could be systematically varied in evaluations. However, varying only five of these elements (content of feedback, intensity of feedback, method of delivery, duration and context) produces 288 combinations [1]. This is before any replication of studies or the addition of other potential elements of an intervention or different modes of delivery of interventions, such as educational meetings or outreach visits. Given the multiplicity of factors that would need to be addressed, such a 'hit and miss' approach is highly inefficient.

The assumption that clinical practice is a form of human behaviour and can be described in terms of general theories relating to human behaviour offers a basis for systematically developing implementation interventions. For example, if there is empirical evidence that a clinical behaviour is influenced by factors such as health professionals' beliefs or perceived control over their practice, then interventions to change their behaviour could include components that target those factors. The explicit use of theory may offer a number of advantages, such as providing a generalisable framework for predicting and interpreting behaviour, designing interventions and evaluating potential causal mechanisms.

However, theory has not commonly been used in the field of implementation research. Within a review of 235 implementation studies only 53 used theory in any way – to inform study design, develop or design the implementation intervention, and/or describe or measure elements of process for *post hoc *interpretation – and only 14 were explicitly theory-based [6].

This paper describes the development of a theory-based intervention to increase the frequency of a clinical behaviour among mental health professionals: appropriate disclosure of the diagnosis to people with dementia. The early care of people with dementia should ideally involve a sensitive and accurate explanation of the diagnosis, the likely prognosis and possible packages of care [7]. Timely disclosure can facilitate decisions about treatment and allows the opportunity to plan family, fiscal and long term care arrangements. In the UK, multidisciplinary mental health teams (MHTs) for older people are often responsible for these tasks. Yet disclosure practice by healthcare professionals varies widely [8]. Most carers are told the diagnosis but people with dementia themselves are often not told [9]. Indeed, disclosure is less likely in dementia than in other terminal conditions, such as cancer. There is therefore substantial scope for improving professional practice.

## Methods

The intervention was developed within the context of an 'implementation modelling experiment' [10,11]. In this type of study, components of an intervention are systematically varied within a randomised controlled design in a manner that simulates a real situation as much as possible. Interim endpoints (stated behavioural intention and behavioural simulation) are measured rather than changes in actual behaviour or healthcare outcome. Behavioural intention is one potentially modifiable factor that can predict actual behaviour. A meta-analysis of experimental evidence shows that a medium-to-large change in intention leads to a small-to-medium change in behaviour [12]. A recent systematic review of the relationship between clinical behaviours and behavioural intention found that the proportion of variance in behaviour explained by intention was of a similar magnitude to that found in the literature relating to non-health professionals [13]. In this study, the experiment was conducted using a cluster randomised controlled trial design with pre- and post-intervention measures. The intervention components were delivered in a paper-based format posted out to participants (members of MHTs for older people). This also contained a questionnaire to gather post-intervention outcome data. The ten key steps in developing this theory based behavioural intervention are outlined in Table 1. This paper provides a full description of the first seven steps; the further methods and results of the experiment and process evaluation will be reported elsewhere.

**Table 1:** Steps in developing a theory based behavioural intervention

1. Specify target behaviour(s).
2. Select theoretical framework (for empirical investigation at baseline and to assess process).
3. Conduct a predictive study with a (preferably representative) sample drawn from the population of interest, to identify modifiable variables that predict the target behaviour(s) and their means/distributions.
4. From predictive study, choose which variables to target. These variables are the proposed mediators of behaviour change.
5. Map targeted variables onto behaviour change techniques and select techniques that (a) are likely to change the mediator variables and (b) it is feasible to operationalise.
6. Choose appropriate method(s) of delivery of the techniques
7. Operationalise intervention components (techniques) in appropriate combination and order
8. Specify control or comparison conditions.
9. Specify hypotheses regarding outcome and process (mediation), i.e. which outcome and predictor variables targeted by the intervention would change compared with the control conditions.
10. Conduct behavioural modelling experiment based on Steps 1–9.

Note: As part of an iterative process, results from the implementation modelling experiment will provide information for feedback loops that address earlier points in this sequence. This feedback loop permits change, development or refinement of the intervention.

### Step 1: Specification of target behaviours

Appropriate disclosure is a process rather than a single behaviour. Hence, appropriate disclosure can encompass multiple actions taken by mental health professionals, usually over a period of time, tailored to individuals' receptiveness and needs for information. Given the lack of an accepted operational definition of appropriate disclosure, we identified potential key behavioural components based on a literature review, interviews with people with dementia and carers, and a consensus panel including a range of professionals and a patient advocate [14].

For the purposes of this study, we focussed on three specific component clinical behaviours. We (CB, ME, JL, JF and RF) used a Delphi process to select the behaviours based on the following criteria: covering different stages of the disclosure process; the earlier consensus panel rankings; importance to people with dementia and carers; evidence of benefit; and potential for change. We selected the following behaviours:

• Finding out what the patient already knows or suspects about their diagnosis;

• Using the actual words 'dementia' or 'Alzheimer's disease' when talking to the patient;

• Exploring what the diagnosis means to the patient.

The published literature relating to social cognition models of behaviour recommends a systematic way to specify behaviour for the purpose of predictive studies. This requires that the behaviour be defined carefully in terms of its Target, Action, Context and Time, or doing what (A), to whom (T) in what context (C) and at what time (T) [15]. This is known as the 'TACT' principle. The behaviours selected for this implementation modelling experiment were specifiable in terms of this TACT principle. For example, for the behaviour 'finding out what the patient already knows or suspects about their diagnosis', the target is the patient, the action is finding out what the patient knows, the context is the clinical condition (dementia) in which the diagnosis is certain and the time is (implicitly) during a consultation prior to formal disclosure.

### Step 2: Selection of theoretical framework

We selected two theories, the Theory of Planned Behaviour (TPB)[16] and Social Cognitive Theory (SCT) [17], for the following reasons. They have been rigorously evaluated in other settings; they predict behaviour in terms of factors amenable to change (e.g., beliefs, perceived external constraints); and they include non-volitional components that acknowledge that individuals do not always have complete control over their actions. There were economies of measurement involved in using both of these theories because of over-lapping constructs. According to the TPB, the strength of a behavioural intention is predicted by attitudes towards the behaviour (in this case, one of the three disclosure behaviours), subjective norms based on the perceived views of other individuals or groups (i.e. perceived social pressure); and perceived behavioural control, encompassing beliefs about self-efficacy (confidence that one can perform an action and that performing the action will have the desired consequence) and wider environmental factors that facilitate or inhibit performance [16]. We further distinguished between attitudes that reflected perceived consequences, for the patient or professional, of doing the behaviour (e.g. whether a disclosure behaviour was harmful or beneficial to the patient) and "emotional attitudes" (e.g. discomfort felt by professionals whilst enacting disclosure behaviours) [18]. SCT considers self-efficacy, outcome expectancy (an individual's estimate that a given behaviour will lead to certain outcomes) and individuals' goals in explaining behaviour, including proximal goals (such as intentions) [17]. We had originally specified Implementation Intentions as a further theory that we intended to operationalise [11,19]. However, the logistical challenges of operationalising the TPB and SCT with this target group were such that Implementation Intentions was dropped to reduce the complexity and length of the intervention package.

Within the context of the implementation modelling experiment, the measurement of potential mediators of behaviour change targeted by the intervention contributes to the process evaluation. Therefore the framework would usually be the same as that used for the initial predictive study. In this case we planned to use TPB and SCT to identify potential causal pathways underlying change so that if change occurred we could explain why [20].

### Step 3: Conduct of a predictive study

We surveyed members of MHTs for older people to identify factors that predict behavioural intentions to follow the three key disclosure behaviours [14]. These are summarised in Table 2. Because the (SCT) outcome expectancy construct actually consisted of all the (TPB) attitude items, for simplicity from this point onwards we will refer to attitudes to cover both of these constructs.

**Table 2:** Summary of regression analyses for the three disclosure behaviours with intention as dependent variable.

**Behaviour**	**Model**	**Variables predicting intention**	**Standardised regression coefficient**	**Change in R^2^(%)**
**Explore what patient already knows or suspects (n = 373)**	TPB constructs	Subjective norm	0.424	23.5***
		Perceived behavioural control	0.161	4.0***
		Emotional attitude	0.094	1.1*
		Attitude	0.098	0.8*
		*Total R*^2^		*29.4*
	SCT constructs	Self efficacy	0.417	22.0***
		Outcome expectancies	0.158	2.2**
		*Total R*^2^		*24.2*
	Team variables	Perceived reliability of colleagues	0.384	13.1***
		Perceived role	0.140	1.2**
		Number of professional groups	-0.113	1.2*
		*Total R*^2^		*15.5*
	Combined constructs	Subjective norm	0.334	23.5***
		Perceived behavioural control	0.213	4.0***
		Perceived reliability of colleagues	0.252	4.6***
		Outcome expectancies	0.145	1.9**
		Number of professional groups responsible for behaviour	-0.128	1.6**
		*Total R*^2^		*35.6*
**Use explicit terminology (n = 366)**	TPB constructs	Subjective norm	0.407	38.8***
		Attitude	0.374	13.1***
		Emotional attitude	0.143	1.8***
		*Total R*^2^		*53.7*
	SCT constructs	Outcome expectancies	0.470	40.1***
		Self efficacy	0.316	7.4***
		*Total R*^2^		*47.5*
	Team variables	Perceived reliability of colleagues	0.566	37.6***
		Perceived role	0.220	4.6***
		*Total R*^2^		*42.2*
	Combined constructs	Outcome expectancies	0.422	40.1***
		Perceived reliability of colleagues	0.284	15.8***
		Subjective norm	0.183	3.7***
		Self efficacy	0.154	1.8***
		Perceived role	0.127	1.3***
		Emotional attitude	-0.133	0.8**
		*Total R*^2^		*63.5*
**Explore what diagnosis means to patient (n = 371)**	TPB constructs	Subjective norm	0.434	36.4***
		Perceived behavioural control	0.389	12.2***
		*Total R*^2^		*48.6*
	SCT constructs	Self efficacy	0.470	29.4***
		Outcome expectancies	0.149	1.7**
		*Total R*^2^		*31.1*
	Team variables	Perceived reliability of colleagues	0.349	10.8***
		Perceived role	0.269	7.2***
		*Total R*^2^		*18.0*
	Combined constructs	Subjective norm	0.334	36.3***
		Perceived behavioural control	0.296	12.3***
		Self efficacy	0.161	1.9***
		Perceived role	0.127	1.2**
		Perceived reliability of colleagues	0.109	1.0**
		*Total R*^2^		*52.7*

* p < 0.05; ** p < 0.01; *** p < 0.001. These significance levels are associated with the increase in R^2 ^as explanatory variables are added stepwise to the regression models. The variable that explained the greatest amount of variation in intention was added first. On subsequent steps the variable that explained the greatest amount of the residual variation was added provided that the improvement in the fit of the model was significant at the 5% level

As the above theories are concerned with individual behaviour, we also added three exploratory, non-theory based questions on perceived roles within the teams on the basis that perceived role may influence intention to perform disclosure behaviours. We were aware that perceived roles and responsibilities for the three behaviours might vary between different professional groups, e.g. giving the actual diagnosis might be perceived as a primary role of the psychiatrist but not (say) the occupational therapist. We therefore added 'team factor' variables to explore their contributions to variation in intention. The questions concerned whether respondents believed each behaviour was his/her responsibility and the perceived reliability of colleagues in performing each behaviour.

### Step 4: Choosing which variables to target for change

In general this decision is empirically driven and includes a consideration of which predictor variables are modifiable. In the current study, the theoretical framework included only modifiable variables. For example, past behaviour may be a strong predictor of current behaviour but, as it is not modifiable, the level of prediction does not inform an intervention to change behaviour. We selected the significant predictors of intention (Table 2) as the variables to target with the intervention.

### Step 5: Mapping of targeted variables on to behaviour change techniques

We used a tool that is the preliminary result of a consensus process involving a collaboration of psychologists and implementation researchers to answer the question, 'which behaviour change technique (out of 35 candidate techniques) is it best to use, in order to change each potential mediator of behaviour change?' [21]. Many techniques included in this tool are themselves theory-based and there is considerable evidence of their effectiveness. For example, well documented ways to improve self-efficacy include 'behavioural modelling' (demonstration of the behaviour) and working through a set of 'graded tasks' (starting with an easy task that a person is confident about, and then gradually increasing task difficulty). The evidence base relating to other change techniques is less clear so the consensus process depended more on participants' own views of the evidence and experience using the techniques. Although this consensus process provided an imperfect evidence base for our mapping process, it is nevertheless the most systematic guide available [21]. We used the consensus-derived tool described by this paper to structure our own deliberations and maximise the possibility of identifying techniques most likely to influence the variables targeted for change.

### Step 6: Choice of appropriate method(s) of delivery of behaviour change techniques

We used an interactive paper-based format posted out to potential participants. We judged that this would represent, within the context of an implementation modelling experiment, the most efficient means of delivering the techniques because a relatively large number of participants could be recruited this way (hence helping to maximise statistical power).

### Step 7: Operationalisation of intervention components

The paper-based mode of delivery of the intervention further influenced the selection and construction of the intervention components, which had to be adapted to this printed format. We therefore discarded a number of potential behaviour change techniques that could not be feasibly applied to a printed format (e.g. motivational interviewing for self-efficacy).

Different ways of operationalising the methods were worked out on an iterative basis involving study team members (MJ, JF, RF, CB, JL & ME). We initially developed intervention methods for one behaviour (finding out what the patient already knows or suspects). Recognising the risk that a paper-based format might be a relatively passive means of delivering the intervention components, we set out to maximise the interactive nature of each section, e.g. by incorporating questions and prompts so that participants had to make active choices when working through it. We undertook cognitive interviews with a convenience sample of three mental health professionals to assess comprehension and acceptability. We then modified the intervention and addressed the other two behaviours.

We did not use separate intervention components to address all predictors of three target behaviours for the following reasons. Firstly, we did not agree upon an acceptable means of operationalising a method to target emotional attitudes. Secondly, incorporating similar types of methods across all three behaviours would result in a lengthy and repetitive instrument. We therefore targeted all three behaviours together – referring to them as 'disclosure of dementia'. We also ensured that the intervention placed more emphasis on targeting one behaviour (use of actual words 'dementia' or 'Alzheimer's disease') that we judged as being particularly pivotal and was associated with most scope for change in intention. The pre-intervention survey had demonstrated that mean behavioural intention significantly differed between the three behaviours, being highest for exploring what the patient already knows or suspects (5.72 on a 1–7 scale) and lowest for the use of explicit terminology (4.66) [14].

Further cognitive interviews were conducted with six other mental health professionals and the intervention was further modified. The final intervention is described next (and available as Additional file 1).

## Results

This section describes the results of this intervention development process. The predictor variables were targeted using a combination of specific behaviour change techniques: persuasive communication; modelling; graded task; and action planning. The full intervention materials are available as an appendix. Table 3 summarises the main techniques used to target each predictive variable. These were as follows.

**Table 3:** Summary of change techniques used to target predictor variables by disclosure behaviour.

**Behaviour targeted**	**Predictor variable targeted**	**Method used to influence behaviour**
Finding out what the patient already knows or suspects	Subjective norm	Persuasive communication
	Self-efficacy	Modelling
Using the actual words 'dementia' or 'Alzheimer's disease'	Subjective norm	Persuasive communication
	Self-efficacy	Modelling
	Self-efficacy	Graded task
	Self-efficacy	Action planning
Exploring what diagnosis means to patient	Subjective norm	Persuasive communication
	Self-efficacy	Modelling
Overall disclosure behaviour	Subjective norm	Persuasive communication
	Beliefs about consequences (attitude)	Persuasive communication
	Perceived Behavioural Control	Environmental changes

### Persuasive communication

Subjective norm was targeted by three types of persuasive communication and for each of these we asked participants for an active (written) response. First, we provided several statements illustrating reasons other mental health professionals have given for disclosing the diagnosis, e.g. "It enables the patient and carer to plan for the future." Participants were asked which statements they agreed with. Second, we presented results from our pre-intervention (baseline) survey of mental health professionals, emphasizing positive findings related to each behaviour, e.g. "96% agreed that finding out what the patient already knows or suspects, before giving the diagnosis, was beneficial to patients." Participants marked which statements they agreed with. The third persuasive communication mainly targeted attitudes (i.e. perceived consequences of the behaviour for professionals and patients). We provided a series of referenced evidence-based statements around disclosure and asked participants to prioritise and mark the three statements they most agreed with, e.g.

"**Many people with dementia want to know their diagnosis**. Relatively few studies have explored the preferences of people with dementia for disclosure of the diagnosis. The proportion of people with dementia wanting to know their diagnosis varies from 33% to 96% [22,23]."

### Modelling

We used modelling to target self-efficacy for each of the three behaviours separately. We did this in the context of a relatively straightforward scenario: the diagnosis of dementia is certain, a helpful carer is present, the patient has insight and the professional has sufficient time. The actual behavioural modelling technique consisted of providing examples of phrases and approaches which other professionals found useful during the disclosure process. For example, for exploring what the diagnosis means to the patient, we suggested: "Do you know anyone else with Alzheimer's Disease or other types of dementia?" Participants could tick boxes beside those that they already used or would consider using, as well as write examples of their own preferred approaches.

### Graded task

The graded task component aimed to help participants achieve incrementally greater levels of 'mastery' by building on existing abilities. It specifically addressed 'using the actual words' because there was most scope for change around this behaviour. We asked participants whether they could confidently use 'the actual words' in five situations of increasing difficulty. For example, it would be easiest to do in the straightforward scenario given for the modelling component. Progressively more difficult situations (initially based on findings from pre-intervention questionnaire and modified following responses during cognitive interviews) ranged from absence of the carer through to the presence of a carer who was interfering (e.g. interrupting the patient). If participants felt that they could confidently 'use the actual words' in all five situations, we asked them to note down a situation where they would find it difficult. Out of the situations participants judged that they would find it difficult to 'use the actual words', we asked them to select the least difficult out of these.

### Action planning

Action planning involves giving a written undertaking to do something specific. Following the graded task, participants were asked to imagine themselves in the least difficult situation and list all possible approaches (which could draw upon any from the modelling component) that could help them before selecting the one they would prefer to use.

### Environmental changes

The final intervention component aimed to address perceived factors in the team environment that might influence disclosure behaviours. This part of the intervention presented participants with examples of approaches used by other teams – often around the ways that local teams or services are organised – that could improve the process of disclosure. For example, this could include: "Mental health teams agreeing a standard process or pathway for the management of people with dementia that specifies steps and responsibilities around disclosure." Participants were asked which of these they used already, thought were useful and thought could be done by their own team.

## Discussion

We have demonstrated the feasibility of systematically developing the major components of a theory-based intervention to change professional practice. This has included the critical steps of identifying variables predictive of behavioural intention and linking them to evidence based methods of behaviour change. Although several previous evaluations have used a theoretical framework, the further development of behaviour change interventions has rarely been underpinned by empirical data on predictors of the behaviour in question.

The explicit use of such approaches and their reporting are frequently absent from published evaluations [6]. Such descriptions, or 'audit trails' of intervention development, can enhance the reproducibility of successful interventions. Furthermore, they help us to identify what it is that is replicated. In the study reported here, there may be a range of lay views about how to describe the key components of the intervention: it could be described as a set of paper-based tasks; provision of information about behaviour; a workbook; brainstorming; a team functioning intervention; or a task analysis intervention. We have proposed that intervention components be described in terms of discrete and identifiable behaviour change techniques (e.g. persuasive communication; action planning) for which there is an existing evidence base of effectiveness in other settings. It would then be possible to investigate whether the same change techniques differ in effectiveness across different modes of delivery (e.g. paper-based versus face-to-face group sessions), without confusing mode of delivery with the intervention content. We argue that this approach to intervention development and reporting is 'scientific' in that it enables us to understand the processes underlying change, to replicate interventions and possibly to improve the delivery of an intervention without altering its essential content. As such, this approach can contribute to a cumulative science of implementation.

There were three main limitations to this work. First, the selection and design of behaviour change techniques was constrained by the mode of delivery (paper-based format); this resulted in challenges around operationalising certain components (e.g. the graded task). For example, we could not find a way to incorporate an intervention method to target one of the predictor variables (emotional attitude) and we decided to target individual cognitive variables rather than team-level variables, as individual variables were consistently stronger predictors of the three target behaviours. It is also possible that some of the intervention methods could be delivered more effectively via other means, e.g. interactive workshops. Second, further compromises arose from the need to keep the paper-based intervention to an acceptable length by either prioritising a targeted behaviour ('using the actual words') or targeting the 'global' behaviour of dementia disclosure. The former meant that we were unable to target all behaviours comprehensively whilst the latter undermines the TACT principle because disclosure is a complex, multi-faceted process [15]. These may have weakened the effectiveness of the intervention components. Third, there was also a risk that, during the detailed design of the intervention methods, we applied some intervention methods to constructs not predictive of targeted behaviours. For example, if the intervention methods are mapped back to targeted constructs (Table 3), we find that PBC for 'using the actual words' was targeted even though this variable was not predictive of intention for this behaviour. Fourth, for the subsequent modelling experiment, more than one behaviour change technique was used to target each of the three behaviours. Hence, we cannot be certain whether any effect on a given variable could directly attributable to a given technique, e.g. whether graded tasks would influence only self-efficacy or whether self-efficacy would be influenced only by graded tasks. We had considered an experimental design whereby participants would be randomly allocated to different techniques for each of the three behaviours and discarded this on the grounds that the study would have insufficient power to detect such effects. However, these limitations are practical issues arising from considerations of feasibility and are likely to influence all intervention development processes.

The UK Medical Research Council Framework for the development and evaluation of complex interventions outlines key phases in intervention development [24]. We have described the steps we took to develop a complex intervention to change professional behaviour, corresponding most closely to the theoretical and early modelling phase of the Framework. However, there were numerous steps that required detailing. We recommend that these steps be considered for the development and reporting of other complex interventions in this field.

This work has underlined further research needs. The process of intervention building we describe requires a strong theoretical and empirical basis, particularly the stage involving mapping predictive constructs on to behaviour change techniques. The behaviour change techniques were also developed for a paper-based method of delivery; further investigation is required to evaluate the relative utility of other methods of delivering and exploring the potential effectiveness of behaviour change techniques, such as interactive computer programmes or workshops. Furthermore, we recognise that targeting people with (for example) low self-efficacy and thereby increasing self-efficacy may not necessarily bring about a change in intention. Nevertheless, it seems sensible to target the variables that predict intention rather than the variable that do not. This is the reason that the interventional modelling experiment approach not only *uses *theory but *tests *theory, by applying an experimental design to assess whether changing variables that predict intention results in changing intention. We see this theory-testing function as a particular strength of intervention modelling experiments.

## Conclusion

It is feasible to develop an intervention to change professional behaviour that is based upon theoretical models, empirical data and evidence based behaviour change techniques. However, achieving an adequate theoretical and empirical basis for intervention development requires systematic work in behaviour change theory and methods, e.g. mapping of constructs on to change techniques. The next step – for the intervention described in this paper – is to evaluate its effects on the constructs that are proposed to mediate behaviour change, and on behavioural intention.

## List of abbreviations

MHT: Mental health team;

SCT: Social Cognitive Theory;

TACT: Target, Action, Context and Time;

TPB: Theory of Planned Behaviour.

## Competing interests

The author(s) declare that they have no competing interests.

## Authors' contributions

ME, MJ, RF, CB, JF & JG designed the study. CB, ME, JL, JF and RF participated in the Delphi process to select the behaviours for the study. MJ, JF, RF, CB, JL & ME conducted the survey work and developed the intervention. CB, JL and RF pre-tested the intervention materials. RF wrote the first draft which was revised by JF and then all other members of the study team. All authors have read and approved the final manuscript.

## Pre-publication history

The pre-publication history for this paper can be accessed here:



## Supplementary Material

Additional file 1 The theory-based intervention. The content and format of the intervention developed in this study. Click here for file
