# Supplementary material for: The development of a theory-based intervention to promote appropriate disclosure of a diagnosis of dementia
Source: BMC Health Serv Res. 2007 Dec 19;7:207. doi: 10.1186/1472-6963-7-207 (PMC2211296; doi:10.1186/1472-6963-7-207)
Supplement: Additional file 1 — The theory-based intervention. The content and format of the intervention developed in this study. [file 1472-6963-7-207-S1.doc]

**Talking to people with dementia about their diagnosis – can we do it better?**

Thank you for continuing to help us with this research study. We are exploring ways to improve the disclosure of the diagnosis to people with dementia. This involves randomly allocating members of mental health teams to receive different types of written materials and asking them to work through a number of scenarios and questions.

We have piloted these materials with your colleagues to check that they are sufficiently interesting and acceptable (e.g. not too time consuming) to complete.

We recognise that the disclosure of dementia can be a complex process involving a number of different steps and people. Whether you are involved in the early or later steps, the tasks and scenarios should still relate to your interactions with patients.

Please now work through this booklet in order, starting with the tasks and ending with the questions about your current practice. Depending on which materials you have been randomly allocated to, everything should take about 15 to 20 minutes to complete. Although you will have seen many of these questions before, we need to ask you them again to help make comparisons. As with the questionnaire you completed at an earlier date, this is not a test. For most questions there are no right or wrong answers and all responses will be treated in the strictest confidence. Some of the questions may seem repetitive or similar; it is important that you try to answer every question.

**We hope that the findings of this study will help improve**

**the quality of care for people with dementia**

**Thank you very much for your time**

If you wish to find out more about this study please contact us:

| Jan Lecouturier  Research Associate | 0191 222 5629 | [Jan.Lecouturier@ncl.ac.uk](mailto:Jan.Lecouturier@ncl.ac.uk) |
| --- | --- | --- |
| Claire Bamford  Senior Research Associate | 0191 222 7047 | [c.h.bamford@ncl.ac.uk](mailto:c.h.bamford@ncl.ac.uk) |
| Robbie Foy  Senior Lecturer in Primary Care | 0191 222 7214 | [R.C.Foy@ncl.ac.uk](mailto:R.Foy@ncl.ac.uk) |

**Here are some reasons that other mental health professionals have suggested for appropriately disclosing the diagnosis to patients with dementia**

*Please* ***tick each box*** *to show which statements you generally agree with*

| |  | | --- | |  | | The patient has a right to know (or not to know) |
| --- | --- | --- | --- |
|  |  |
| |  | | --- | | It helps to avoid later confusion and ambiguity |
|  |  |
| |  | | --- | | To identify the starting point for sharing information about the diagnosis and treatment options |
| |  | | --- | | To foster a collaborative relationship between the patient and professional |
|  |  |
| |  | | --- | | It makes future communications with the patient, carers and other professionals easier |
|  |  |
| |  | | --- | | It enables the patient and carer to plan for the future |
|  |  |
| |  | | --- | | To enable the patient to start sorting out legal, financial and practical issues |
|  |  |
| |  | | --- | | It maintains openness in the relationship with the patient |
|  |  |
| |  | | --- | | Other *(please note briefly below)* |
|  |  |
|  |  |
|  |  |

**A recent national survey asked a sample of UK mental health teams for older people about key aspects of disclosing a diagnosis of dementia.**

**There were 368 respondents.**

*Please* ***tick each box*** *if you agree with the views expressed by the mental health teams surveyed*

| |  | | --- | |  | | 96% agreed that finding out what the patient already knows or suspects before giving the diagnosis was beneficial to patients |
| --- | --- | --- | --- |
|  |  |
| |  | | --- | | 91% agreed that exploring the meaning of the diagnosis with the patient was a good use of professionals’ time |
|  |  |
| |  | | --- | | Only 5% thought that using the actual words ‘dementia’ or ‘Alzheimer’s disease’ discouraged patients from planning for the future |
|  |  |

**Here are some statements, supported by research evidence, about the disclosure of dementia**

*Please put a cross in the box beside the* ***THREE*** *statements which you find most persuasive.*

| |  | | --- | |  | | **Many people with dementia want to know their diagnosis.** Relatively few studies have explored the preferences of people with dementia for disclosure of the diagnosis. The proportion of people with dementia wanting to know their diagnosis varies from 33% ^1^ to 96% ^2^. |
| --- | --- | --- | --- |
|  |  |
| |  | | --- | | **Most people with dementia thought it helpful to have been told their diagnosis.** Following disclosure, the majority of people with dementia thought it was helpful to have been told their diagnosis (75%) ^3^. |
|  |  |
| |  | | --- | | **People given a diagnosis of dementia develop positive ways to cope with their diagnosis.** Studies of the impacts of disclosure indicate that people with dementia develop positive ways of coping with their diagnosis ^4^; ^5^. Key benefits of disclosure identified by people with dementia include   - validation of their perception that something is wrong ^6^ - being able to make sense of their experience ^7^ |
|  |  |
| |  | | --- | | **Being given a diagnosis confirms some patients’ own suspicions.** For some participants, the diagnosis confirmed their suspicions that they had Alzheimer’s disease^5^. |
|  |  |
| |  | | --- | | **Lack of information can cause distress.** In contrast, lack of information caused distress and forestalled opportunities to engage in grief work to cope with the losses experienced^7^. |
|  |  |
| |  | | --- | | **There is little evidence of catastrophic reactions to being told the diagnosis.** Despite two early reports of suicide following diagnostic disclosure, longitudinal studies have concluded that there is little evidence of catastrophic reactions, including depression, to disclosure^5^. |
|  |  |
| |  | | --- | | **The negative consequences of disclosure are no greater or more persistent than those of other serious diagnoses.** Disclosure of a diagnosis of dementia has been shown to have a number of negative emotional consequences, including shock, anger, fear, depression and denial ^4^, ^5^, ^8^. There is evidence of restriction of activities following disclosure and negative impacts on self esteem ^4^, ^8^, ^9^. There is, however, no evidence that these negative consequences of disclosure are any greater or more persistent than those associated with the disclosure of other serious diagnoses. |
|  |  |

(1) Marzanski M. Would you like to know what is wrong with you? On telling the truth to patients with dementia. J Med Ethics 2000; 26:108-113.

(2) Dautzenberg PLJ, van Marum RJ, van der Hammen R, Paling HA. Patients and families desire a patient to be told the diagnosis of dementia: a survey by questionnaire on a Dutch memory clinic. Int J Geriatr Psychiatry 2003; 18:777-779.

(3) Jha A, Tabet N, Orrell M. To tell or not to tell - comparison of older patients' reaction to their diagnosis of dementia and depression. Int J Geriatr Psychiatry 2001; 16:879-885.

(4) Pratt R, Wilkinson H. A psychosocial model of understanding the experience of receiving a diagnosis of dementia. Dementia 2003; 2(2):181-191.

(5) Young RF. Medical experiences and concerns of people with Alzheimer's disease. In: Harris PB (ed). The person with Alzheimer's disease: pathways to understanding the experience. Baltimore & London: The Johns Hopkins University Press; 2002. 29-46.

(6) Robinson P, Ekman S-L, Wahlund L-O. Unsettled, uncertain and striving to understand: toward an understanding of the situation of persons with suspected dementia. Int J Aging Hum Dev 1998; 47(2):143-161.

(7) McWilliams E. The process of giving and receiving of a diagnosis of dementia: an in-depth study of sufferers', carers' and consultants' experiences. PSIGE Newsletter 1998; 64(March):18-25.

(8) Husband HJ. The psychological consequences of learning a diagnosis of dementia: three case examples. Aging Ment Health 1999; 3(2):179-183.

(9) Husband HJ. Diagnostic disclosure in dementia: an opportunity for intervention? Int J Geriatr Psychiatry 2000; 15:544-547.

Disclosing the diagnosis of dementia can be relatively straightforward or very difficult, depending on circumstances and the individual patient. Here, we would like you to focus on a relatively straightforward situation. **For this, assume that you are certain of the diagnosis, a helpful carer is present, the patient has insight and you have sufficient time.** (You will consider more difficult situations later.)

Below are some approaches and examples of phrases which other professionals find useful. Please tick the boxes for those that you already use or think you might want to use. They also don’t need to be the *exact* approaches or words you would use. Tick as many boxes as you like.

| *General approach* | *Examples of specific phrases or actions* | ***Already use*** | ***Might want to use*** |
| --- | --- | --- | --- |
| **Finding out what the patient already knows or suspects** | “Do you have any concerns about what has been happening to you recently?” | |  | | --- | | |  | | --- | |
|  | “What do you think could possibly be causing your memory problems?” | \|  \| \| --- \| | \|  \| \| --- \| |
|  | “What is your biggest fear about what might be causing your problems?” | \|  \| \| --- \| | \|  \| \| --- \| |
| *You or your colleagues might have other preferences. If so please briefly note them here:* | | | |
| **Using the actual words ‘dementia’ or ‘Alzheimer’s Disease’ when talking to the patient**  *NB. Depending on roles within your team, this might relate to first making a diagnosis or to later appointments or visits.* | “I think there is a serious cause for your memory problems...” | |  | | --- | | |  | | --- | |
|  | “The things that have been happening to you recently are probably because of dementia / Alzheimer’s Disease.” | \|  \| \| --- \| | \|  \| \| --- \| |
|  | “I have the results of your recent tests. The results look like those we’d get for people with dementia / Alzheimer’s Disease.” | \|  \| \| --- \| | \|  \| \| --- \| |
| *You or your colleagues might have other preferences. If so please briefly note them here:* | | | |
| **Exploring what the diagnosis means to the patient** | “I don’t know whether you already know this, but Alzheimer’s Disease is one type of dementia.” | |  | | --- | | |  | | --- | |
|  | “Do you know anyone else with Alzheimer’s Disease or other types of dementia?” (As a means of starting discussion about prognosis) | \|  \| \| --- \| | \|  \| \| --- \| |
|  | “Have you had any thoughts or concerns about practical things that might affect you in the future, such as money or driving?” | \|  \| \| --- \| | \|  \| \| --- \| |
| *You or your colleagues might have other preferences. If so please briefly note them here:* | | | |

This section is about using the actual words ‘dementia’ or ‘Alzheimer’s Disease’ when talking to patients. Depending on your own role within your team, this might take place after first making a diagnosis or during subsequent appointments or visits.

Five situations now follow. These have been ranked from easiest to most difficult based upon the experience of other mental health professionals.

**Starting with number 1, consider each situation separately in turn** and place a **tick in the box** to indicate how confident you are that you could use the actual words ‘dementia’ or ‘Alzheimer’s Disease’ when talking to the patient.

| **Could you confidently use the actual words ‘dementia’ or ‘Alzheimer’s Disease’ when talking to the patient (and you are certain of the diagnosis) if …** | | **Yes** | **No** | **Maybe** |
| --- | --- | --- | --- | --- |
| **1** | … a helpful carer is present, the patient has insight and you have sufficient time? | |  | | --- | | |  | | --- | | |  | | --- | |
|  |  |  |  |  |
| **2** | … the carer or relative is absent, the patient has insight and you have sufficient time? | |  | | --- | | |  | | --- | | |  | | --- | |
|  |  |  |  |  |
| **3** | … a helpful carer is present, the patient has insight, and you are short of time? | |  | | --- | | |  | | --- | | |  | | --- | |
|  |  |  |  |  |
| **4** | … a helpful carer is present, the patient lacks insight and you have sufficient time? | |  | | --- | | |  | | --- | | |  | | --- | |
|  |  |  |  |  |
| **5** | … a carer or relative is present but is interfering with communication, e.g. interrupting the patient, the patient has insight and you have sufficient time? | |  | | --- | | |  | | --- | | |  | | --- | |

If **ALL** your responses are **YES…**

If you have responded **NO** or **MAYBE** for **any** of the situations above …

Can you think of a situation in which you would find it difficult to use the words ‘dementia’ or ‘Alzheimer’s Disease’ when talking to the patient?

Briefly describe the situation below.

**Now go to the next page**

Select from the **NO’s/MAYBE’s** which one you find the **LEAST** difficult

Write the number (1-5) of this situation in the box below

**Now go to the next page**

**This section is about the situation you chose (or described) on the previous page**

Imagine yourself with a patient in the situation you have selected or described on the previous page.

**Firstly,** record a list of up to five possible alternative approaches that would help you in that situation.

| **Possible alternative approaches** |  |
| --- | --- |
|  | 1 |
|  | 2 |
|  | 3 |
|  | 4 |
|  | 5 |

**Secondly**, now try to visualise your chosen situation. Based on your responses immediately above, which approach do you plan to use?

When it next arises I plan to use approach:

Please circle all that apply

| 1 | 2 | 3 | 4 | 5 |
| --- | --- | --- | --- | --- |

Other mental health professionals have suggested that the following approaches – often around the ways that local teams or services are organised - might help improve the process of disclosing a diagnosis of dementia.

Out of this list, there are some approaches that you might already use, you might think are useful and could use. You may tick more than one box for each approach.

| **Approaches that might help improve the process of disclosing a diagnosis of dementia** | Please tick if you … | | |
| --- | --- | --- | --- |
|  | **… use this already** | …think this is **useful** | … (and your team) **could** do this |
| Mental health teams agreeing a standard process or pathway for the management of people with dementia that specifies steps and responsibilities around disclosure |  |  |  |
| Having structured case records and communication materials, e.g. using standardised formats for recording issues discussed with patient and for clinic letters |  |  |  |
| Having patients prepared for consultations, e.g. by sending out written information about what to expect |  |  |  |
| Scheduling sufficiently long appointment times for patient consultations |  |  |  |
| Ensuring that during appointments, patients and carers have an opportunity for a private discussion with you. For example, by co-working with a colleague, allowing you to talk to the patient whilst your colleague talks to the carer |  |  |  |
| Offering written information following consultations |  |  |  |
| Educating referrers on what happens in clinics and what to tell patients (e.g. educational meetings or referral guidelines for GPs) |  |  |  |
| Offering pre-diagnostic counselling, e.g. about potential outcomes of any diagnostic tests |  |  |  |
| Having a patient advocate (e.g. from the Alzheimer’s Society) present during consultations |  |  |  |
| ***Please add any other approaches that you already use or wish to suggest:*** |  |  |  |
